# Facing Pediatric Rheumatic Diseases: The Role of Disease Activity and Parental Emotion Regulation Strategy in Parents’ and Children’s Psychological Adjustment

**DOI:** 10.3390/children10121863

**Published:** 2023-11-28

**Authors:** Emanuela Del Giudice, Riccardo Lubrano, Sonia Monique Bramanti, Alessandra Babore, Carmen Trumello, Sara Giovanna De Maria, Anna Dilillo, Alessia Marcellino, Vanessa Martucci, Mariateresa Sanseviero, Silvia Bloise, Flavia Ventriglia, Valerio Manippa

**Affiliations:** 1 Pediatric and Neonatology Unit, Maternal and Child Department, Sapienza University of Rome, Polo Pontino, 04100 Latina, Italy; 2 Department of Psychological, Health and Territorial Sciences, University “G. d’Annunzio” of Chieti-Pescara, 66100 Chieti, Italy; 3 Department of Education, Psychology and Communication, University of Bari Aldo Moro, 70122 Bari, Italy

**Keywords:** pediatric rheumatic diseases, parental emotion regulation strategy, children’s psychological adjustment, children’s emotional difficulties, behavioral difficulties, chronic and recurrent pain

## Abstract

Background: Pediatric rheumatic diseases (PRDs) are a group of chronic disorders that start in childhood and are characterized by periodic exacerbations and remissions of symptoms, with limitations in family, school, and social activities. The aim of this study was to detect differences in parents’ psychological adjustment and emotion regulation strategies, and parent-reported children’s adjustments in families of children with active and inactive PRDs. Methods: Fifty-four parents (38 mothers and 16 fathers) of children with PRD were recruited from a pediatric unit. Disease activity was evaluated by their pediatric rheumatologist, while parents’ depressive and anxiety symptoms, emotion regulation strategies, and children’s emotional difficulties and hyperactivity–inattention symptoms were assessed through a web-based survey. Results: Parents of children with active PRDs reported higher levels of their child’s emotional difficulties and hyperactivity–inattention symptoms. Linear regression analysis demonstrated that having a child in the active phases of PRD and lower use of cognitive reappraisal lead to higher children’s emotional symptoms, while active disease, low use of cognitive reappraisal, and greater expressive suppression were associated with higher hyperactivity–inattention symptoms. Our study highlights that children with PRDs and their parents may be at increased risk for psychological problems, especially during the active disease phase, highlighting the importance of a multidisciplinary approach.

## 1. Introduction

Caring for a child with a chronic condition puts the parents in front of a unique set of stressors related to the illness and its treatment [1] that make parents more vulnerable to experiencing anxiety and depression and could result in poor adherence to medical treatment, a worse parent–child relationship, and greater difficulties for children [2]. 

Pediatric rheumatic diseases (PRDs) comprise a group of rare chronic disorders that originate in childhood and may persist into adulthood. They are characterized by a dysfunctional immune response that can cause inflammation in various organs [3]. Juvenile idiopathic arthritis (JIA) is the most common PRD and affects approximately 1 in every 1000 children, with an incidence of 11 to 14 new cases per 100,000 children [4,5]. PRD symptomatology is characterized by a relapsing–remitting activity that may vary over time and includes pain, fatigue, and disability, which may also be associated with potential comorbidities [6,7] that can restrict family life, school attendance, and social activities [8]. Disease activity, in the context of rheumatic disease, is a multidimensional measure that refers to the symptoms related to inflammation and may fluctuate widely during the patient’s disease course [9], influencing the physical and biological functioning of children with PRDs and their psychological well-being and social life [10].

PRDs may have a physical and psychosocial impact on both affected children and their parents [11]. As for children, previous studies have highlighted higher levels of emotional difficulties and conduct problems in children with PRDs when compared with healthy ones [12,13]. In addition, experiencing negative emotions can lead to perceiving greater pain and functional disability [14]. With regards to parents, the unpredictable and chronic nature of these diseases could lead to increased depressive and anxious symptoms [15]. 

In this context, parental emotion regulation has particular importance because it facilitates the acceptance of the disease by both parents and children [16]. In fact, parents with high levels of distress and who tend to use maladaptive regulation strategies, such as rumination or expressive suppression, can affect the psychological well-being of their affected children, their adaptation to the disease, and consequently their levels of pain and disability [17].

Starting from these premises, our first aim was to detect differences in parents’ anxious and depressive symptoms and emotion regulation strategies (i.e., cognitive reappraisal and expressive suppression) and parent-reported children’s adjustment (i.e., emotional difficulties and hyperactivity–inattention symptoms) in families of children with active PRDs and inactive PRDs. Furthermore, we analyzed if and to what extent parents’ emotion regulation strategies and children’s disease activity state were associated with children’s difficulties. 

## 2. Materials and Methods

### 2.1. Participants and Study Design

We performed a cross-sectional study conducted in a monocentric center via a web-based survey, developed using Qualtrics XM, in May–June 2021. Parents of PRD patients referred to the Pediatric Unit of Santa Maria Goretti Hospital, Latina—Sapienza University of Rome (Polo Pontino) were invited to participate. We had informed 48 participants about the study, and among them, 39 gave their consent to participate.

The total sample comprised 54 Italian parents (38 mothers and 16 fathers) of 39 children (21 females) affected by PRD. Inclusion criteria were having a child diagnosed with PRD, according to international criteria, including JIA [18], autoinflammatory [19], and connective tissue diseases [3,20]. Exclusion criteria included: presence of mental disorders in the parents; children with any associated conditions such as malignancy; history of chronic metabolic, infectious, and other systemic diseases; and food allergies, malabsorption, or obesity [body mass index (BMI) ≥ 30]. 

Parents were informed about the purpose and procedures of the study, and each gave his/her consent by clicking “Yes, I accept to participate in the study.” In addition, during the first medical examination, informed consent was obtained by all participants. All procedures were fully compliant with the Declaration of Helsinki and with the Ethics Code of the Italian Board of Psychology. This study was approved by the Institutional Review Board of the Department of Psychological, Health, and Territorial Sciences of the University “G. d’Annunzio” of Chieti. 

### 2.2. Socio-Demographic and Medical Screening

We created an ad hoc questionnaire to assess parents’ and children’s demographic data. Children’s disease-related variables such as disease type, age at disease onset, disease duration, medical treatments, and disease activity parameters were assessed at the time of the survey by the same pediatric rheumatologist who usually follows all patients. Particularly, each patient was screened for disease activity using the physician global assessment of disease activity (medical doctor (MD) global) expressed on a 21-circled scale (0–10 in increments of 0.5, where 0 is no activity and 10 is maximum activity, with inactive disease if the score was zero and active disease in the case of a score higher than 0) from a routine blood sample. All JIA patients were assigned to active or inactive PRD groups according to MD global score [21].

### 2.3. Psychological Measures

The Hospital Anxiety and Depression Scale (HADS) [22,23] is a self-reported tool designed to assess anxious and depressive symptomatology among individuals in a non-psychiatric sample. It was divided into an “Anxiety” subscale (sample item: “I feel tense or wound up”) and a “Depression” subscale (sample item: “I still enjoy the things I used to enjoy”), both containing seven intermingled items. All items were rated on a four-point Likert scale ranging from 0 to 3, with different statements for each point depending on the item request. A higher score indicates greater levels of anxiety or depression. Cronbach’s alpha for anxiety and depression subscales was, respectively, 0.86 and 0.74.

The Emotion Regulation Questionnaire (ERQ) [24,25] is a self-reported tool designed to assess two specific emotion regulation strategies that are typically used to manage positive and negative emotions in daily life: expressive suppression and cognitive reappraisal. The cognitive reappraisal scale is composed of six items (sample item: “When I’m faced with a stressful situation, I make myself think about it in a way that helps me stay calm”) evaluating the cognitive ability to modify the meaning and the emotional impact of a situation. The expressive suppression scale is composed of four items (sample item: “I keep my emotions to myself”) evaluating the inhibition of emotion-expressive behavior. All items were rated on a seven-point Likert scale ranging from 1 (“strongly disagree”) to 7 (“strongly agree”). A higher score indicates greater use of that specific emotion regulation strategy. Cronbach’s alpha was 0.78.

The Strengths and Difficulties Questionnaire (SDQ) [26,27] is administered to parents to evaluate their perception of their children’s emotional and behavioral problems. For our aims, we selected 2 subscales out of 5: the emotional symptoms (sample item: “Many fears, easily scared”) and hyperactivity–inattention (sample item: “Easily distracted, concentration wanders”) subscales made up of 5 items, in which each participant responds on a 3-point Likert scale ranging from 0 (“not true”) to 2 (“certainly true”). Scores on each scale range from 0 to 10, with a higher score indicating that parents perceive higher levels of emotional and hyperactivity–inattention symptoms in their child. Specifically, in the emotional symptoms and hyperactivity–inattention scales, respectively, scores above 5 and 6 indicate very high emotional difficulties and greater hyperactivity symptoms. Cronbach’s alpha was 0.79.

### 2.4. Statistical Analysis

All data analyses were conducted using the IBM Statistical Package for the Social Sciences (SPSS, Version 19). The significance level for all analyses was set at *p* < 0.05. The selection of the appropriate statistical tests for each variable was determined after testing for normal distribution with the Kolmogorov–Smirnov normality test. According to the variables, categories, and distribution, appropriate descriptive statistics were performed to investigate participants’ sociodemographic characteristics and medical assessment of children’s disease. Particularly, independent sample comparisons were performed by using Pearson Chi-square tests for categorical variables (e.g., sex and marital status distributions), Student’s *t*-test for parametric continuous variables (e.g., age of children and disease duration), and the Mann–Whitney U Test for non-parametric continuous variables (i.e., PGA of disease activity).

Since our main dependent variables were all normally distributed, a series of independent sample Student *t*-tests were run to test for differences between active PRD vs. inactive PRD groups in parents’ emotion regulation strategies (i.e., cognitive reappraisal, expressive suppression), anxiety, depression, and parent-reported children’s difficulties (i.e., emotional symptoms and hyperactivity–inattention). Finally, a series of linear regression analyses were performed to test the effects of parental emotion regulation (i.e., cognitive reappraisal and expressive suppression) and disease activity during study enrollment on children’s difficulties (i.e., emotional symptoms and hyperactivity–inattention). In the regression model, disease activity was coded as a dummy variable (1 = active PRD; 0 = inactive PRD). 

## 3. Results

As regards children’s clinical characteristics, 24 were affected by JIA, 8 by autoinflammatory diseases (of whom 6 had periodic fever, aphthous stomatitis, pharyngitis, adenitis; 1 had familial Mediterranean fever, and 1 had Behcet’s disease), and 7 by connective tissue diseases (of whom 5 had systemic lupus erythematosus, 1 had Sjögren’s disease, and 1 had juvenile dermatomyositis). Moreover, 44% of our sample had comorbidities such as uveitis, allergies, and thyroiditis. Overall, 27 patients were on therapy (25.9% self-administered medications).

At the time of enrollment, 12 were parents of children in the active phase of the disease (i.e., active PRD) and 42 were parents of children in the inactive phase of the disease (i.e., inactive PRD). The M age of parents was 45.5 (SD = 5.7) years, whereas their children had a M age of 13.9 (SD = 5.0) years. The baseline characteristics of patients and their parents are reported in Table 1.

**Figure 1:**
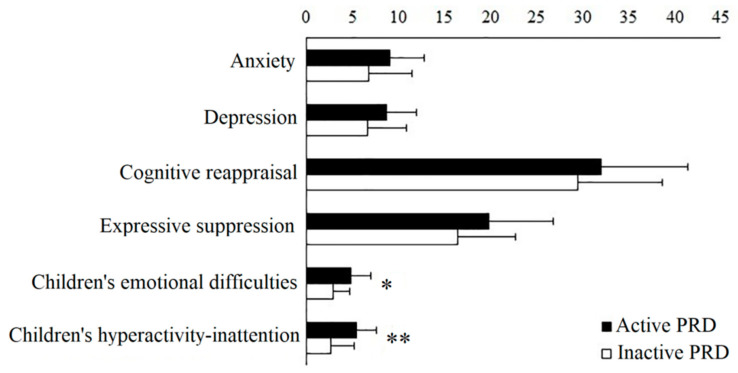
Mean and standard deviation scores of the self-reported scales administered to parents of children with active or inactive PRD. Note. Anxiety and depression are assessed through the Hospital Anxiety and Depression Scale. The cognitive reappraisal and expressive suppression scales of the Emotion Regulation Questionnaire assessed the strategies used by the parents to manage emotions. The emotional symptoms and the hyperactivity–inattention scales of the Strengths and Difficulties Questionnaire evaluate the parent-reported children’s emotional difficulties and hyperactivity–inattention symptoms. ** *p* < 0.001. * *p* < 0.01. Abbreviation: PRD, pediatric rheumatic diseases.

Parents of children who were in an active phase of PRD during the enrollment reported higher levels of their child’s emotional (*p* = 0.005) and hyperactivity–inattention (*p* = 0.001) symptoms. No differences emerged in anxiety, depression, and emotion regulation strategies between parents of children with active and parents of children with inactive PRD (see Figure 1).

A series of linear regressions were performed to detect the association between parents’ emotion regulation strategies (i.e., cognitive reappraisal and expressive suppression) and children’s disease activity and children’s emotional and hyperactivity symptoms. Findings showed (Table 2) that cognitive reappraisal (*p* = 0.023) and the presence of active PRDs (*p* = 0.002) were related to children’s emotional symptoms, with 19% of variance explained. 

The tendency of parents to adopt expressive suppression was not found to be related to children’s emotional symptoms. Hence, low parental cognitive reappraisal and having a child in an active phase of the PRD led parents to perceive and report higher emotional symptoms in children. As for children’s hyperactivity symptoms, active PRD (*p* < 0.001), parental cognitive reappraisal (*p* < 0.001), and expressive suppression were significant predictors (*p* = 0.036), accounting for the 37% of variance. Thus, having a child in an active phase of PRD and a low use of cognitive reappraisal in favor of a large use of expressive suppression led parents to perceive and report higher levels of children’s hyperactivity.

## 4. Discussion

Chronic illnesses, especially in childhood, inevitably involve the entire family system, causing repercussions on daily life, school, and social activities, and may affect the well-being of parents and children. In particular, PRDs are characterized by symptoms of exacerbation (active PRD) and remission (inactive PRD) that could result in different psychological reactions in both parents and children [15,28,29]. Our data showed that parents’ psychological adjustment was not associated with disease activity, whereas children’s higher emotional and hyperactivity–inattention symptoms were reported by parents of children with active PRDs compared to parents of children with inactive PRDs.

Although existing research has described higher levels of anxiety and depression in parents of children with active PRDs [15,30] as compared with parents of children with inactive PRDs, we did not observe such a difference in our sample. In line with a previous study [31], parents of children with a chronic disorder, such as PRDs, could be more vulnerable to negative psychological outcomes, such as anxiety and depression, independent of the disease activity status, as they have to constantly manage many physical and psychological challenges related to the disease, resulting in greater emotional and behavioral difficulties for their children. Moreover, restrictions in daily life activities and the need to manage and administer specific medical treatments, as well as the associated side effects, can significantly impact parental wellbeing [31,32]. This influence on wellbeing may persist, even during inactive disease phases, potentially resulting in an absence of discernible differences in anxiety and depression levels between parents of children with active disease and those with inactive disease states. This underscores the pivotal role of parents’ perceptions of the disease’s status and progression. 

In addition, parents with children in the active phase of a PRD reported both higher emotional and hyperactivity–inattention symptoms in their children compared to parents of inactive PRD patients. These findings seem to support the hypothesis that the physical and practical challenges faced by children with PRD are exacerbated during the active phase of the disease, resulting in an increase in children’s emotional and behavioral difficulties. Previous studies involving samples of adult patients with rheumatic disease have identified higher levels of anxiety and depression in patients in the active phase of the disease [33,34]. Studies carried out in children with PRD are partially in agreement with our results, identifying only greater emotional difficulties and not differences in terms of behavioral problems [29,35]. It is plausible that certain illness-related factors, which tend to be more pronounced during the active phase of the disease, may contribute to heightened levels of frustration and increased somatic complaints among pediatric patients. These factors, in turn, may lead to elevated levels of both emotional and behavioral issues. Notably, the impact on emotional problems appears to be more substantial [36], as the physical limitations imposed by the disease restrict outward behavioral expressions [29]. Moreover, it is possible that externalized symptoms are more difficult to detect since they depend on a complex interaction of social, familial, and physical factors [35], which are already limited due to the disease, and which could be more limited during the active phase.

Additionally, we analyzed if and to what extent disease activity state and parents’ emotion regulation strategies were associated with children’s difficulties. Results from the linear regression analysis showed that having a child in active phases of PRD and parents’ lower use of cognitive reappraisal led to higher levels of children’s emotional symptoms. Additionally, active disease states, low use of cognitive reappraisal, and higher levels of expressive suppression were associated with higher levels of children’s hyperactivity–inattention symptoms. The unpredictable nature of this chronic condition, characterized by pain and fatigue, may influence children’s vulnerability perception during active disease phases, resulting in greater emotional difficulties and hyperactivity symptoms [18]. Additionally, the unpredictable fluctuations in the disease and concerns about its progression [36] can heighten parents’ sensitivity to changes in their children’s physical and emotional wellbeing [15]. 

Regarding emotion regulation strategies, they refer to the ability to evaluate, monitor, and modify emotional reactions to stressful events. In order to regulate emotions, a wide range of cognitive, behavioral, and physiological processes are required [25]. Parental emotion regulation helps children to understand, display, and regulate their own emotions [37]. In the context of chronic pediatric disease, the illness represents a significant source of chronic stress that could contribute to emotional and behavioral problems in children and can compromise adherence to medical treatment regimens [38]. Parents in this context may be more likely to develop more adaptive emotion regulation strategies for stressors and traumatic events to deal with the chronicity of their children’s illness, functioning as a protective factor for their psychological maladjustment [12] and children’s difficulties.

Finally, our research revealed that parents’ cognitive reappraisal plays a crucial role in ameliorating children’s emotional difficulties and hyperactivity–inattention symptoms, while parents’ expressive suppression is primarily associated with heightened hyperactivity–inattention symptoms. This seems to suggest that a greater utilization of adaptive emotion regulation strategies, such as cognitive reappraisal, within the parental context can promote a family environment that fosters the open expression of emotions and facilitates effective communication among family members [16]. Such an environment is linked to improved psychological outcomes, as it serves to mitigate emotional and behavioral challenges in both parents and children [16] and enhances their ability to cope with issues related to illness. Conversely, the use of maladaptive strategies, such as emotional suppression, particularly in the context of chronic disease [38], is linked to unfavorable psychological outcomes. This may be attributed to parents’ proclivity to inhibit the expression of their emotions, consequently fostering a family environment in which children do not feel encouraged to freely express their own emotions. As a result, this may contribute to heightened externalizing of problems, exemplified by increased hyperactivity.

### Limitations and Future Directions

Caution is needed in interpreting our findings, as our study is not without limitations. First of all, since PRDs are rare diseases affecting 1 child in 1000 [4], our sample size comprised a decreased number of patients and parents, and this may limit the generalizability of results. Moreover, due to the heterogeneity of our sample, which consisted of different rheumatological diseases, we did not use more disease-specific scores. In addition, the cross-sectional and monocentric nature of the research design and the unbalancing between the active and inactive PRD samples prevented us from drawing conclusions as to the directionality of the associations. Furthermore, all measures concerning parents’ and children’s psychological adjustment were self-reported, and other instruments, both implicit and explicit, could provide different outcomes. Although data were collected one year after the start of the COVID-19 pandemic, we did not analyze its impact, as the focus was mainly on children’s disease activity and parents’ emotion regulation strategies, variables less likely to be subjected to contingent events, such as the COVID-19 pandemic. Finally, children’s difficulties relied on the parents’ self-report, which may not necessarily align with the children’s psychological wellbeing. Future longitudinal studies should consider employing a more extensive sample size that encompasses both parents and children in order to comprehensively address these issues.

## 5. Conclusions

The current study contributes to the limited body of literature on this underexplored topic, shedding light on the potential heightened risk of psychological difficulties among children with PRD and their families. Specifically, our study indicates that active disease status, an increased tendency among parents to employ expressive suppression, and a decreased use of cognitive reappraisal as emotion regulation strategies by parents are associated with a greater likelihood of emotional and hyperactivity issues in these pediatric patients. 

These results emphasize the critical need for mental health screening and tailored psychological support for pediatric patients with PRD and their parents, particularly when the disease is in an active phase. Such an approach could allow for a more comprehensive understanding and integration of the emotional experiences and perspectives of both patients and children within a multidisciplinary therapeutic framework.

## Figures and Tables

**Table 1:** Demographic and medical characteristics of the study sample. Asterisks indicate significant comparisons (*p* < 0.05).

	Total Sample (*n* = 54)	Active PRD (*n* = 12)	Inactive PRD (*n* = 42)	Comparisons by Group (Active PRD vs. Inactive PRD)
Parent characteristics				
Age in years (M ± SD)	45.50 ± 5.755	48 ± 5.268	44.53 ± 5.796	*p* = 0.065
Sex				*p* = 0.715
Male	16 (30%)	3 (25%)	13 (31%)	
Female	38 (70%)	9 (75%)	29 (69%)	
Marital status				*p* = 0.208
Relationship	47 (87%)	9 (75%)	38 (90%)	
No relationship	7 (13%)	3 (25%)	4 (10%)	
Education				*p* = 0.648
Middle school or less	22 (40%)	6 (50%)	16 (38%)	
High school or equivalent	21 (39%)	5 (42%)	16 (38%)	
College degree or higher	11 (21%)	1 (8%)	10 (23%)	
Household income (euros/year)				*p* = 0.022 *
0–15,000	22 (41%)	6 (50%)	16 (38%)	
<15,001–28,000	23 (42%)	4 (33%)	19 (45%)	
28,001–55,000	7 (13%)	0 (0%)	7 (17%)	
>750,000	2 (4%)	2 (17%)	0 (0%)	
Patient characteristics				
Age of children (in years) (M ± SD)	13.90 ± 5.036	15.89 ± 3.296	13.30 ± 5.351	*p* = 0.446
Child’s sex				*p* = 0.318
Male	18 (%)	4 (44%)	14 (47%)	
Female	21 (%)	5 (56%)	16 (53%)	
PGA of Disease Activity (Me, IQR)	0, 0–5	6, 5–7	0, 0–0	*p* < 0.001 *
Disease (duration in years) (M ± SD)	6.67 ± 4.276	5.56 ± 3.67	7 ± 4.44	*p* = 0.309
Comorbidity				*p* = 0.515
Yes	17 (44%)	6 (77%)	11 (37%)	
No	22 (56%)	3 (33%)	19 (63%)	
Medical treatment				*p* = 0.115
cDMARDs	10 (26%)	3 (33%)	7 (23%)	
bDMARDs	7 (18%)	2 (22%)	5 (17%)	
csDMARD plus bDMARD	10 (26%)	4 (45%)	6 (20%)	
No treatment	12 (30%)	0 (0%)	12 (40%)	

Abbreviations: PRD, pediatric rheumatic disease; M, mean; SD, standard deviation; PGA, Physician Global Assessment; Me, median; IQR, interquartile range; csDMARDs, conventional synthetic disease-modifying anti-rheumatic drugs; bDMARDs, biologic disease-modifying antirheumatic drugs.

**Table 2:** Regression analysis for children’s emotional difficulties and hyperactivity–inattention symptoms reported by parents (dependent variable).

	R = 0.476; R^2^ =0.227; Adjusted R^2^ = 0.197					R = 0.637; R^2^ = 0.406; Adjusted R^2^ = 0.370				
	F (2,53) = 7.490; *p* < 0.001					F (3,53) = 11,389; *p* < 0.001				
Variables	Emotional Difficulties					Hyperactivity–Inattention				
	B	SE	β	t	*p* Level	B	SE	Β	t	*p* Level
Disease activity	1.993	0.600	0.411	3.320	=0.002	2.769	0.724	0.428	3.827	<0.001
Cognitive reappraisal	−0.064	0.027	−0.291	−2.352	=0.023	−0.137	0.033	−0.464	−4.128	<0.001
Expressive suppression			0.200	1.569	=0.217	0.102	0.047	0.246	2.150	=0.036

## Data Availability

The datasets analyzed during the current study are available from the corresponding author upon reasonable request. The data are not publicly available due to privacy and ethical restrictions.

## Abbreviations

PRD: pediatric rheumatic disease; JIA, juvenile idiopathic arthritis; BMI, body mass index; HADS, Hospital Anxiety and Depression Scale; ERQ, Emotion Regulation Questionnaire; SDQ, Strengths and Difficulties Questionnaire; M, mean; SD, standard deviation; PGA, Physician Global Assessment; Me, median; IQR, interquartile range; csDMARDs, conventional synthetic disease-modifying anti-rheumatic drugs; bDMARDs, biologic disease-modifying antirheumatic drugs.
